# Effect of *Allium sativum* and *Nigella sativa* on alleviating aluminum toxicity state in the albino rats

**DOI:** 10.3389/fvets.2022.1042640

**Published:** 2022-11-29

**Authors:** Sayed Soliman Abdel Ghfar, Montaser Elsayed Ali, Maha Abdullah Momenah, Fatimah A. Al-Saeed, Amin A. Al-Doaiss, Yasser Sabry Mostafa, Ahmed Ezzat Ahmed, Mohamed Abdelrahman

**Affiliations:** ^1^Department of Animal Productions, Faculty of Agriculture, Al-Azhar University, Cairo, Egypt; ^2^Department of Animal Productions, Faculty of Agriculture, Al-Azhar University, Assiut, Egypt; ^3^Department of Biology, College of Science, Princess Nourah bint Abdulrahman University, Riyadh, Saudi Arabia; ^4^Biology Department, College of Science, King Khalid University, Abha, Saudi Arabia; ^5^Department of Theriogenology, Obstetrics, and Artificial Insemination, Faculty of Veterinary Medicine, South Valley University, Qena, Egypt; ^6^Key Lab of Agricultural Animal Genetics, Breeding and Reproduction of Ministry of Education, Huazhong Agricultural University, Wuhan, China; ^7^Animal Production Department, Faculty of Agriculture, Assiut University, Asyut, Egypt

**Keywords:** aluminum toxicity, *Allium sativum*, *Nigella sativa*, histopathological abnormalities, liver, kidney, testes

## Abstract

The study objective was to evaluate *Allium sativum's* potential and *Nigella Sativa's* combination's potential to reduce aluminum toxicity and return to the normal state. In the present study, a hundred albino rats were randomly divided into five equal groups. The first group was used as a control group; the other four groups were exposed to aluminum 1,600 ppm. The second exposed to aluminum only; the third and fourth groups were treated with *Allium sativum* 5% and *Nigella sativa* 5%, respectively, while the fifth group was treated with a mix of *Allium sativum* 2.5% and *Nigella sativa* 2.5% for 8th weeks. After 8 weeks, the aluminum administration was stopped, and the second group was divided into three groups. The groups were treated with *Allium sativum* 5% and *Nigella sativa* 5%, and a mix of *Allium sativum* 2.5% and *Nigella sativa* 2.5%, respectively. The first group was the control group (continued from the first experiment). Garlic and *Nigella sativa* were crushed and added to feed while receiving aluminum chloride daily at a dose of 1.6 ml/l was added to the drinking water. Histopathological changes in the liver, kidney, and testes were investigated after 8 and 16 weeks, and blood samples were collected after 4, 8, and 16 weeks for biochemical blood parameters. The results showed that the histopathological examination of the liver, kidney, and testes showed signs of congestion in blood vessels after aluminum exposure. Meanwhile, the treatment with *Allium sativum* or *Nigella sativum* or the mixture between them had positive effects on evading the harmful effects of aluminum in the liver, Kidney, and testes tissues. In addition, there were protective effects for *Allium sativum* and *Nigella sativa* against aluminum on serum creatinine, urea, ALT, and AST concentrations. The present study concluded that supplementation *with Allium sativum* or *Nigella sativa* or their combination could reduce aluminum toxicity and return the liver, kidney, and testes to normal.

## Introduction

Aluminum is a trivalent action found in its ionic form in most animal and plant tissues and natural water everywhere (1). It is the third most prevalent element and the most abundant metal in the earth's crust, representing ~8% of total mineral components (2). The sources of Al are especially corn, yellow cheese, salt, herbs, spices, tea, cosmetics, aluminum ware, and containers. Also, Al is widely used in antacid drugs, as well as in food additives and toothpaste (3).

Aluminium's toxic action is to cause oxidative stress by producing reactive free radicals that can overwhelm the cell's antioxidant defenses and cause cellular injuries where the ion interacts with plasma membrane moieties, cytoplasmic biomolecules, mitochondria, and nuclear structures. In contrast, oxidative injuries are caused by the oxidation of proteins, lipids, and nucleotides, which results in the generation of altered functional biomolecules with impaired operational capabilities for cellular homeostasis (4–6).

Aluminum is a neurotoxic agent that causes oxidative stress (7). It is known to cause toxic effects on various systems, including the brain, bone, Kidney, and blood (8). It affects several enzymes and other biomolecules relevant to Alzheimer's disease (9, 10). Plants are the green factors on our planet; they convert carbon dioxide and water to carbohydrates and nitrogen to amino acids. Besides food, plants are considered the natural green pharmacy, providing drugs to maintain good health and restore humans' failed health. The medical arts originated when humankind first began to use remedial measures to eliminate their pains, suffering, and other illnesses (11).

Furthermore, *Nigella sativa*, also known as black seed, black cumin, or habatul Barakah, has long been used in the Middle East as a traditional medicine for various complaints, headache, cough, flatulence, as a choleretic, antispasmodic, uricosuric and have a positive effect of the immune system (12). *Nigella sativa* includes a variety of active ingredients such as nigellicine, nigellimine, nigellidine, and alphahederin, as well as thymoquinone, thymohydroquinone, dithymoquinone, thymol, and carvacrol (13). Also, *Nigella sativa* protects against renal ischemia-reperfusion-induced oxidative injury, nephrotoxicity, and hepatotoxicity induced by disease or chemicals (14).

*Allium sativum* belongs to the family Liliaceae and the genus Allium (15). *Allium sativum* is commonly used in food, and its medical properties have been well-recognized throughout history and ancient civilizations. Traditional medical practitioners have considered *Allium sativum* an excellent medicinal plant with many therapeutic potentials. *Allium sativum* includes secondary metabolites such as flavonoids, polyphenols and tannins, as well as bioactivity such as antibacterial, antifungal, anti-inflammatory, pancreatic-amylase, glucoamylase enzyme inhibitors, antiplatelets and has excellent anti-oxidant properties, which scavenged reactive oxygen species (ROS), enhanced cellular antioxidant enzymes, and increased glutathione in the cells (16, 17).

The present study was designed to study the aluminium's toxic effects on the liver, kidney, testis, and some blood measurements and to study the ability of medicinal plants to treat this effect and to return the organs to their normal states after the exposure period.

## Materials and methods

### Study location

This study was conducted at the animal house laboratory, animal production department, faculty of agriculture, Al-Azaher University, Cairo, Egypt, located on the Nile River, 160 km (100 miles) inland from the Mediterranean Sea and 135 km (80 miles) west of the Red Sea, latitude 27.18°N, and longitude 31.19°E.

### Animals and experimental design

One hundred male albino rats, apparently healthy and clinically free of diseases, with a body weight of 80–100 g and aged 1.5 months, were included in this study. Rats were obtained from El Osman Farm, Cairo, Egypt, housed in stainless steel cages, and provided with food, water, and *libitum*. Aluminum chloride was received daily at a dose of 1.6 ml/l added to the drinking water (18, 19).

### Exposure to aluminum toxicity

**Figure 1:**
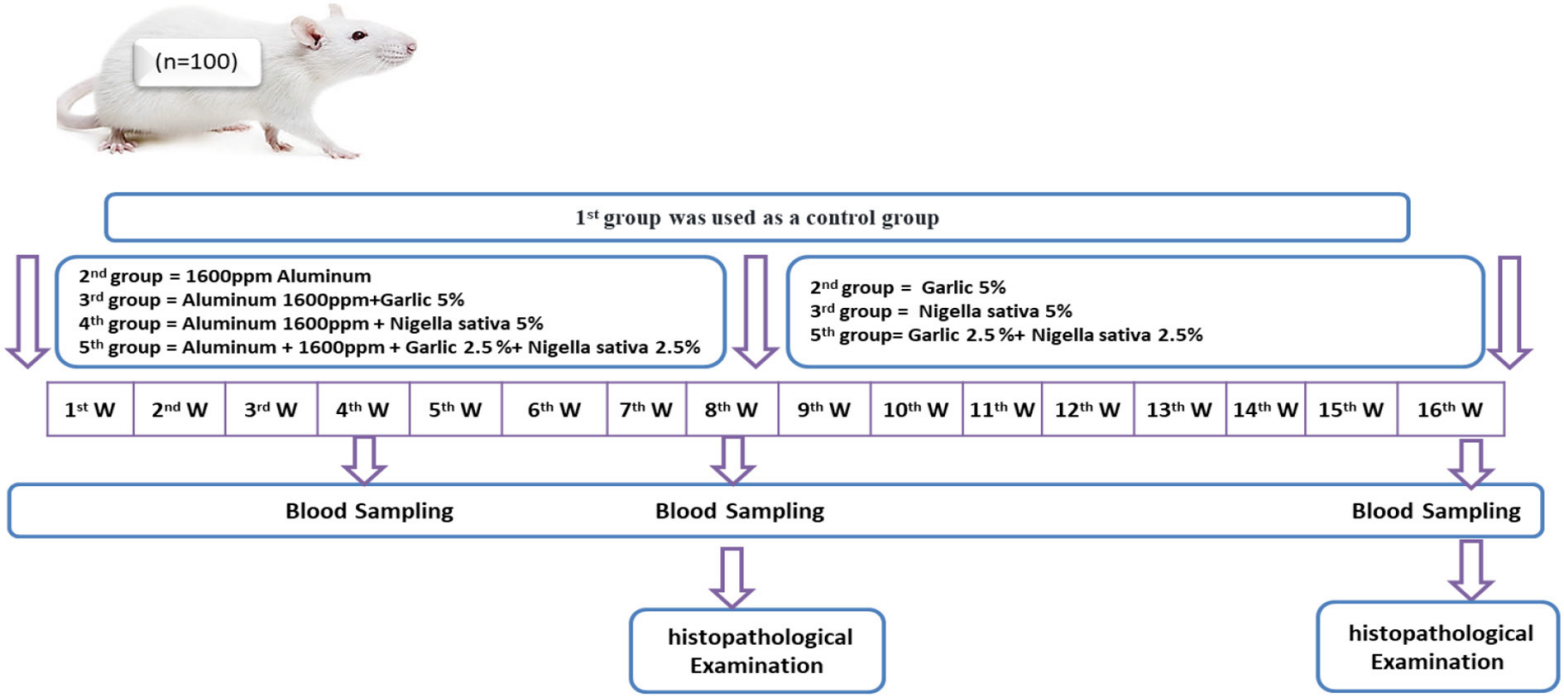
Schematic diagram showing the experimental protocol for the effect of *Allium sativum* and *Nigella sativa* on reducing the negative consequences of Aluminum and returning liver, Kidney, and test to' natural state in the Albino rats.

Rats were randomly divided into five equal groups (20 rats/each); the Control group (G1), Aluminum chloride 1,600 ppm/rat (G2), Aluminum chloride 1,600 ppm + *Allium sativum* 5% (G3), Aluminum chloride 1,600 ppm + *Nigella sativa* 5% (G4), and Aluminum 1,600 ppm + *Allium sativum* 2.5%+ *Nigella sativa* 2.5% (G5) treated groups, for 60 days (Figure 1).

### Return to normal status after exposure to aluminum toxicity

After exposure to aluminum toxicity, for 8 weeks, returning to normal status was examined by subdividing 15 rats into three groups (5 rats/each); the ferst group was saved as the control group (which continued from the first experiment); the second and therd subgroups treated with *Allium sativum* 5% and *Nigella sativa* 5%, respectively; the fourth subgroup treated with a mix of *Allium sativum* 2.5% and *Nigella sativa* 2.5% (Figure 1).

### Histopathological examination of the groups during exposure to aluminum toxicity and return

Histological samples were taken after 8 and 16 weeks. Immediately after collection, the livers, kidneys and testes were immersed in a formalin concentration of 10% for 2 days, washed in water, dehydrated in ascending grade of ethyl alcohol, cleared by xylene, and embedded in melted paraffin wax. The samples were examined by a laboratory microscope (H & E., Stain, X 100). The liver and kidney blocks were sectioned at six-micron cut and stained with eosin and hematoxylin, according to (20).

### Blood sampling

Blood samples were obtained from rats by withdrawing blood from the orbital venous plexuses using a capillary tube at the fourth, eighth, and 16th weeks. Blood samples were centrifuged at 3,000 rpm for 10 min, and then sera and plasma samples were harvested and stored at−20°C until further assays. Serum total protein and albumin were determined according to Burtis (21) and Gindler and Gindler and Westgard (22) using kits supplied by SPINREACT, S Chemical Company, SPAIN. Aspartate transaminase (AST), alanine transaminase (ALT), Creatinine, and urea were determined according to Mathieu et al. (23) and Tabacco et al. (24) using kits supplied by LINEAR CHEMICALS Chemical Company, SPAIN.

### Statistical analysis

Statistical analysis of the presented data was performed using the (25), followed by Duncan's multiple range test (26) to test the effect of Aluminum, *Allium sativum*, and *Nigella sativa* after 4, 8, and 16 weeks of the experiment. The normal distribution of the data was confirmed using the Kolmogorov–Smirnov test. The *p* values ≤ 0.05 of significance were considered.

## Results

### Exposure to aluminum toxicity

#### Histopathological liver

**Figure 2:**
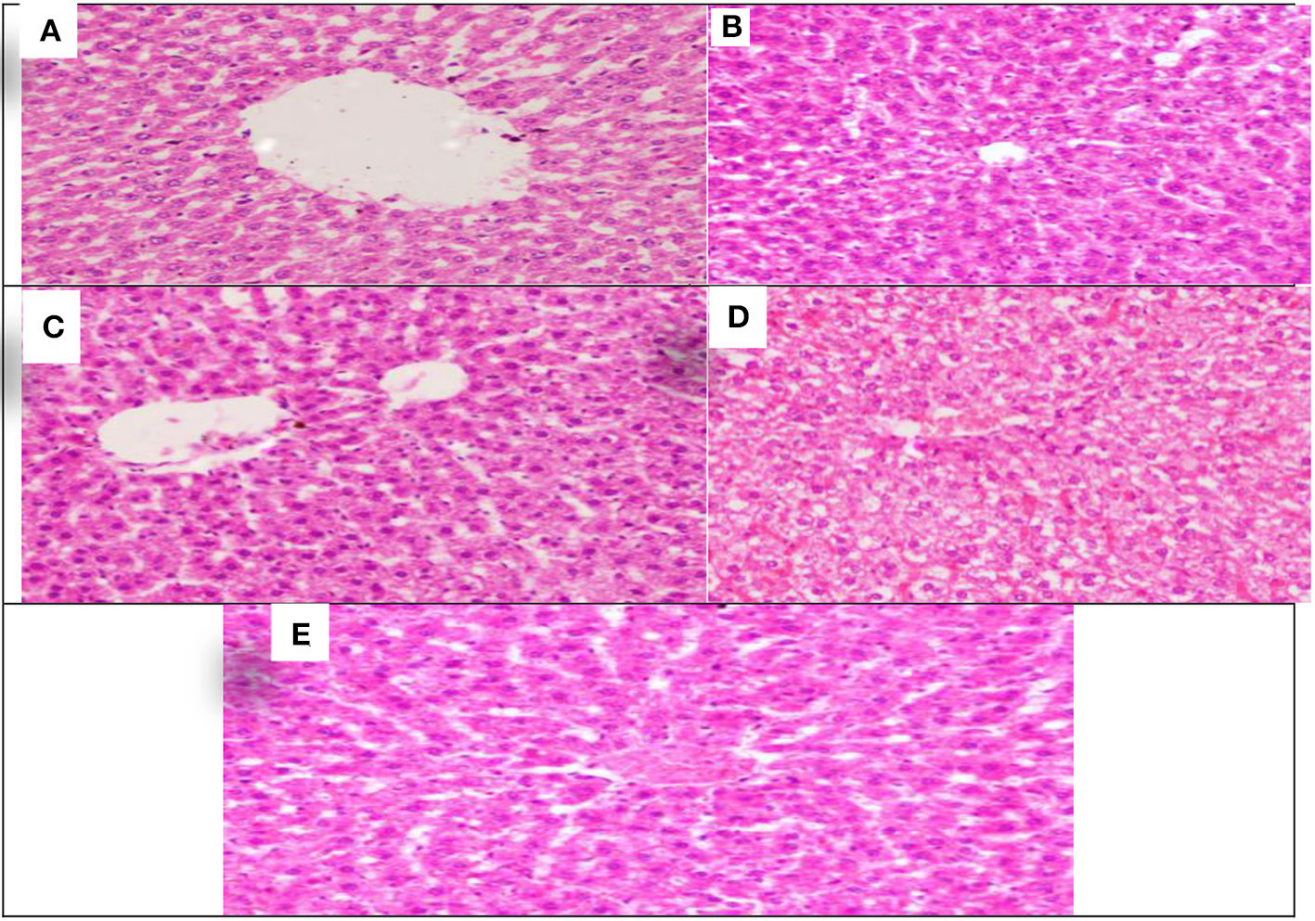
Schematic diagram showing the effect of exposure to aluminum and medicinal plants and their mix on the histopathology of rats' liver at 60 days after treatment. **(A)** Control group. **(B)** 1,600 ppm Aluminum. **(C)** 1,600 ppm Aluminum + *Allium sativum* 5%. **(D)** 1,600 ppm Aluminum + *Nigella sativa* 5%, and **(E)** 1,600 ppm Aluminum + *Allium sativum* 2.5% + *Nigella sativa* 2.5%.

The effect of *Allium sativum* or *Nigella sativa* on the histopathology of albino rats' liver in the 8th week after treatment during aluminum exposure was examined compared to that of the control rats and presented in Figure 2.

Histopathological sections of *Allium sativum* 5%, *Nigella sativa* 5%, the combination, and the control group showed well-maintained lobular structure, portal tracts consisting of the hepatic artery, portal vein, bile duct, the central veins; some were normal and some congested (Figures 2A,C–E). The hepatocytes were normal in arrangement, cytoplasm, and nuclei. Meanwhile, in the aluminum 1,600 ppm treated group (Figure 2B), hepatic sinusoids revealed mild congestion compared with the control group, which showed typical structures for the liver. This indicates that the treatment with *Allium sativum* or *Nigella sativa* or the mixture between them had positive effects on evading the harmful effects of aluminum in liver tissues.

#### Histopathological kidney

**Figure 3:**
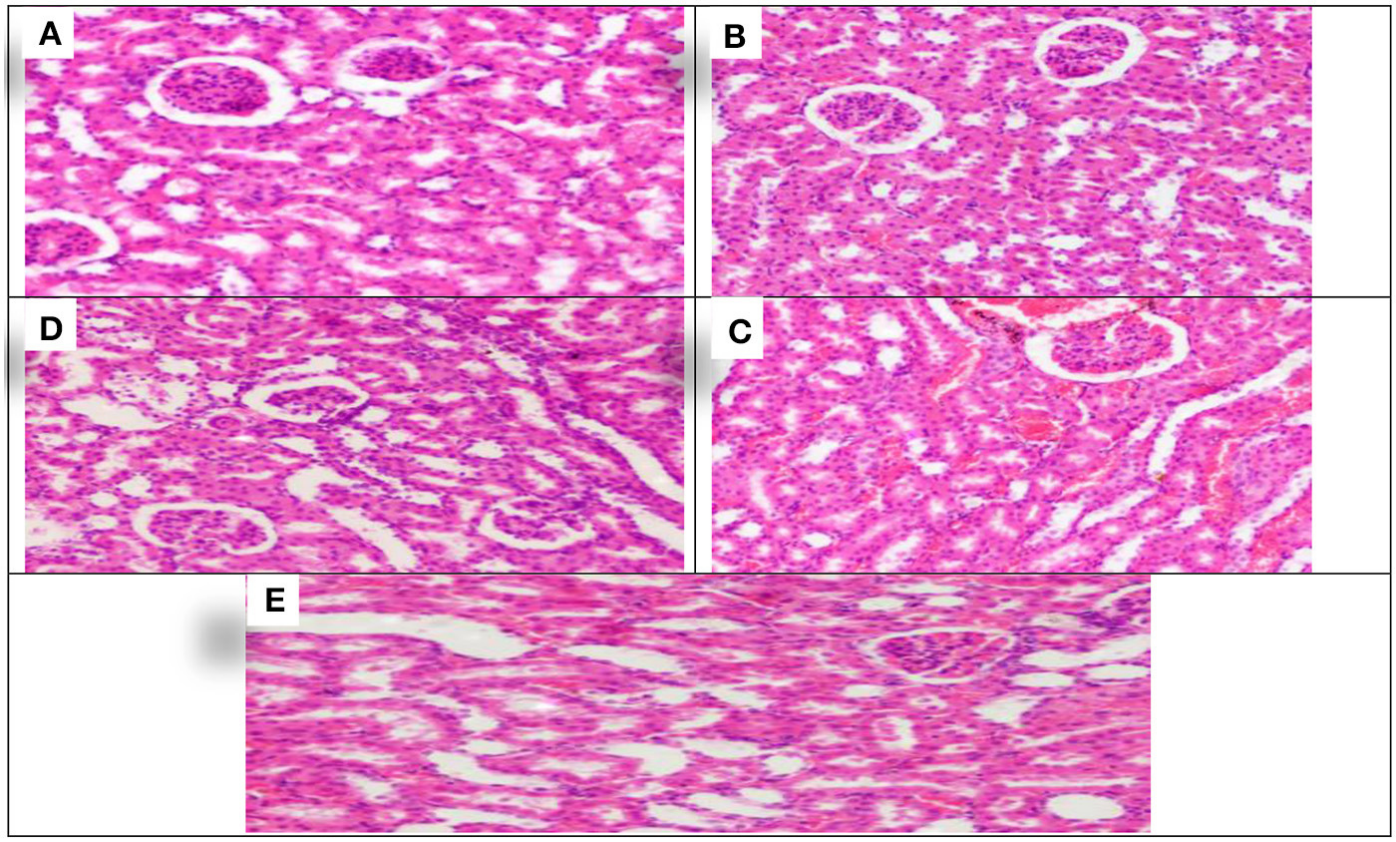
Schematic diagram showing the effect of each aluminum, medicinal plant, and mix with aluminum and medicinal plants on histopathology of rats' Kidney 60 days after treatment. **(A)** Control group. **(B)** 1,600 ppm Aluminum. **(C)** 1,600 ppm Aluminum + *Allium sativum* 5%. **(D)** 1,600 ppm Aluminum + *Nigella sativa* 5%, and **(E)** 1,600 ppm Aluminum + *Allium sativum* 2.5% + *Nigella sativa* 2.5.

The effect of treating with *Allium sativum* and *Nigella sativa* during exposure to aluminum on the kidneys' histopathology after the 8 weeks of treatment was examined in comparison with that of the control rats (Figure 3).

The study showed the effect of aluminum and treatment with *Allium sativum* and *Nigella sativa* on the histopathology of rats' Kidneys compared with the control group. Sections in the kidney tissue showed preserved architecture. The cortex revealed normal glomeruli and tubules. The medulla revealed normal tubules and mildly congested blood vessels of interstitial tissue.

Meanwhile, at 1,600 ppm, aluminum-treated group sections in the kidney tissue showed preserved architecture, and the cortex revealed normal glomeruli and tubules with congestion of the cortical blood vessels (Figure 3B). In comparison, treatment with medicinal plants *Allium sativum* 5%*, Nigella* 5%, and the mixture between them (*Allium sativum* 2.5% + *Nigella* 2.5%), showed average results in Kidney samples. Sections in the kidney tissue showed preserved architecture; the cortex revealed normal glomeruli and tubules, and the medulla revealed normal tubules and mildly congested blood vessels of interstitial tissue. This indicates that the treatment with *Allium sativum* or Nigella or the mixture between them had positive effects on evading the harmful effects of aluminum in Kidney tissues (Figures 3C–E).

#### Histopathological testes

**Figure 4:**
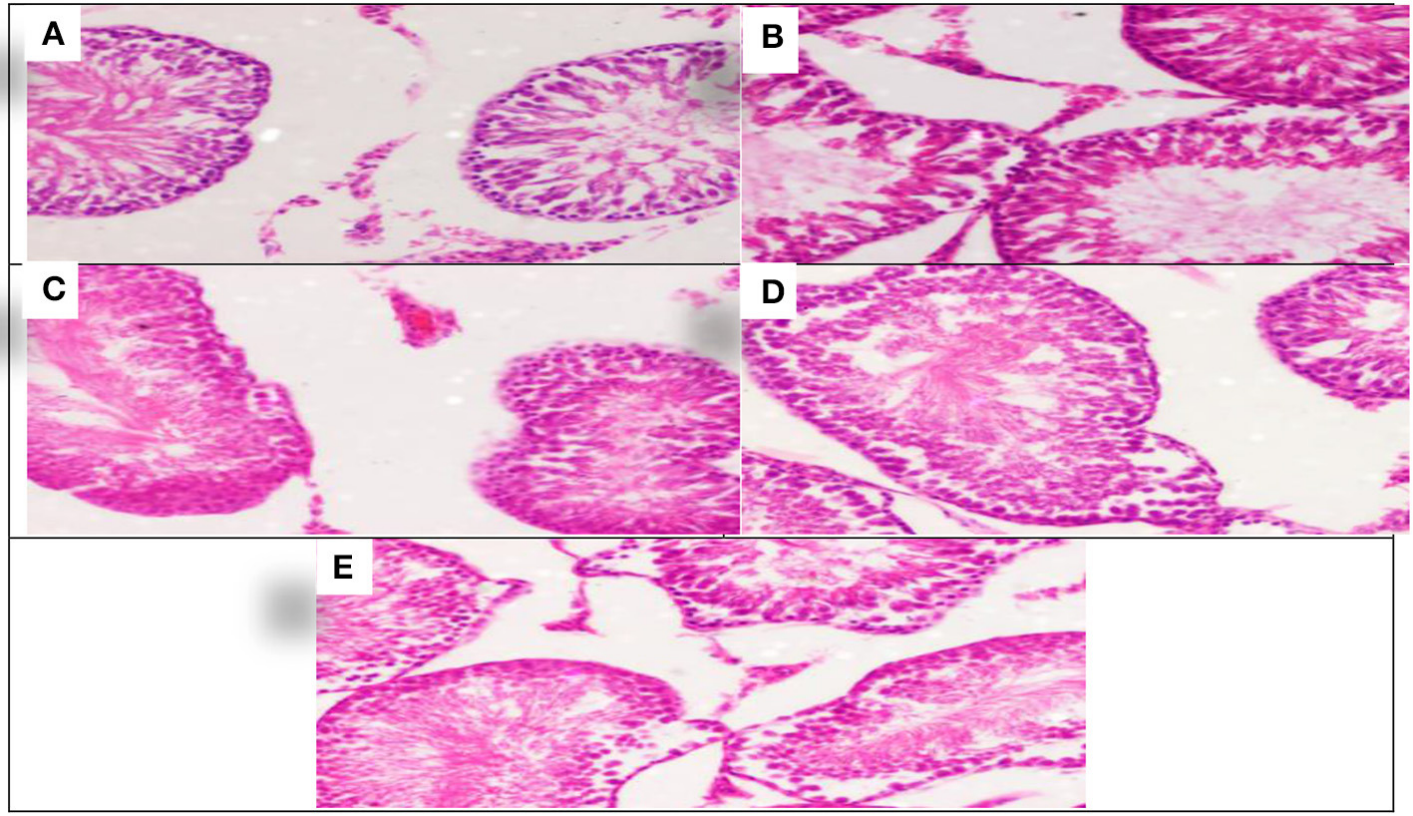
Schematic diagram showing the effect of each aluminum, medicinal plant, and mix with aluminum and medicinal plants on histopathology of rats' Tests 60 days after treatment. **(A)** Control group. **(B)** 1,600 ppm Aluminum. **(C)** 1,600 ppm Aluminum + *Allium sativum* 5%. **(D)** 1,600 ppm Aluminum + *Nigella sativa* 5%, and **(E)** 1,600 ppm Aluminum + *Allium sativum* 2.5% + *Nigella sativa* 2.5%.

The effect of each *Allium sativum* and *Nigella sativa* during exposure to aluminum on the testes histopathology after 8 weeks of treatment were compared to the control rats (Figure 4).

Aluminum, *Allium sativum*, and *Nigella sativa* affect the histopathology of rats' testicular tissue in the 8th week compared with the control group. Sections in the testicular tissue showed preserved histological architecture. The testicular seminiferous tubules consisted of normal basement membrane spermatogonia arranged in a stratified manner, spermatocytes, and spermatozoa in the lumen. However, Sertoli cells were normal in number and arrangement. The testicular tissue structure in all groups, including lobular architecture: normal seminiferous tubules and Sertoli cells, was normal without histological changes (Figures 4A–E).

#### Serum blood biochemical parameters

The results of biochemical blood parameters in control, *Allium sativum*, and *Nigella sativa* at the fourth and 8th week during exposure to aluminum 1,600 ppm toxicity are presented in Table 1.

**Table 1:** Serum Biochemical Parameters (mg//dl) and in control, *Allium sativum* and *Nigella sativa* at fourth and 8th week during exposure to aluminum 1,600 ppm toxicity (*n* = 20/group, mean ± SEM).

**Parameter (mg//dl)**	**Time**	**Control**	**Aluminum^1, 600 ppm^**	**Aluminum** ^**1, 600 ppm**^			**Sig**
				***Alliumsativum* ^5%^**	**N. *Sativa*^5%^**	***Alliumsativum*^2.5%^ + N. *Sativa*^2.5%^**	
Total Protein	4th	8.80 ± 0.10^a^	6.36 ± 0.08^d^	7.65 ± 0.09^c^	7.77 ± 0.07^c^	8.14 ± 0.09^b^	*
	8th	8.74 ± 0.04^a^	6.14 ± 0.04^d^	7.20 ± 0.10^c^	7.34 ± 0.12^c^	8.25 ± 0.08^b^	**
Albumin	4th	5.77 ± 0.03^a^	4.73 ± 0.09c	5.17 ± 0.03^bc^	4.87 ± 0.32^c^	5.63 ± 0.09^b^	***
	8th	5.76 ± 0.12^a^	4.17 ± 0.03^b^	5.54 ± 0.24^a^	4.50 ± 0.21^b^	5.40 ± 0.23^a^	***
Creatinine	4th	0.72 ± 0.01^c^	1.22 ± 0.01^a^	0.94 ± 0.01^b^	0.95 ± 0.02^b^	0.75 ± 0.02^c^	***
	8th	0.78 ± 0.03^b^	1.65 ± 0.16^a^	0.88 ± 0.01^b^	0.96 ± 0.02^b^	0.75 ± 0.02^b^	***
Urea	4th	34.33 ± 1.86^c^	56.00 ± 2.08^a^	43.67 ± 1.76^b^	48.33 ± 2.03^b^	35.00 ± 0.58^c^	***
	8th	33.00 ± 1.73^b^	53.73 ± 3.39^a^	49.33 ± 3.76^a^	52.60 ± 2.33^a^	40.67 ± 1.76^b^	***
ALT	4th	45.33 ± 2.03^b^	57.00 ± 1.53^a^	48.50 ± 0.29^b^	44.00 ± 2.65^b^	44.67 ± 1.20^b^	***
	8th	41.00 ± 0.58^b^	66.00 ± 2.08^a^	43.33 ± 2.33^b^	44.00 ± 2.08^b^	45.67 ± 1.76^b^	***
AST	4th	30.46 ± 1.86^c^	34.33 ± 8.11^a^	35.50 ± 14.43^ab^	32.50 ± 4.04^bc^	31.63 ± 2.96^c^	***
	8th	31.53 ± 1.45^cd^	39.03 ± 2.40^a^	36.00 ± 2.65^b^	32.10 ± 1.53^b^	31.40 ± 1.15^d^	***

a, b, c, d Means with different superscripts in the same row are *Means in the same row differ significantly (*p* ≤ 0.05); **Means in the same row differ significantly (*p* ≤ 0.01); ***Means in the same row differ significantly (*p* ≤ 0.001).

The total protein and albumin concentrations (mg/dl; mean = SEM) at D30 and D60 was significantly decreased (*P* < 0.01) in the aluminum 1,600 ppm treated group, while there were no significant differences between *Allium sativum* 5% and *Nigella Sativa* 5% in the total protein concentration. Additionally, the change in total protein concentration in the animal treated with the *Allium sativum* 2.5% plus *Nigella Sativa* 2.5% was non-significant and approached the control group (Table 1).

The study revealed that treating with *Allium sativum* 5% and *Nigella Sativa* 5% maintained the creatinine concentration (mg/dl; mean = SEM) at the normal level in the fourth and 8th week, and there were no significant (*P* > 0.01) differences between control group at the 8th week. In addition, the animals treated with a mix of medicinal plants (*Allium sativum* 2.5% + *Nigella sativa* 2.5%) had the same creatinine concentration (mg/dl; mean= SEM) as the control group in the fourth and 8th week.

The urea concentration (mg/dl; mean = SEM) had no significant differences between the mix of medicinal plants (*Allium sativum* 2.5% + *Nigella Sativa* 2.5%) treatment and the control group in the fourth and 8th week. In contrast, the urea concentration was significantly higher (*P* < 0.01) in comparison to those of the Control group after the fourth and 8th week.

Regarding liver enzyme function, both AST and ALT levels (mg/dl; mean = SEM) showed significant (*P* < 0.01) higher levels between the aluminum 1,600 ppm treated group and other groups in the fourth and 8th week. However, the treatment with *Allium sativum* and *Nigella sativa* decreased ALT and AST percentages (Table 1).

### Return to normal status after exposure to aluminum toxicity

#### Histopathological liver

**Figure 5:**
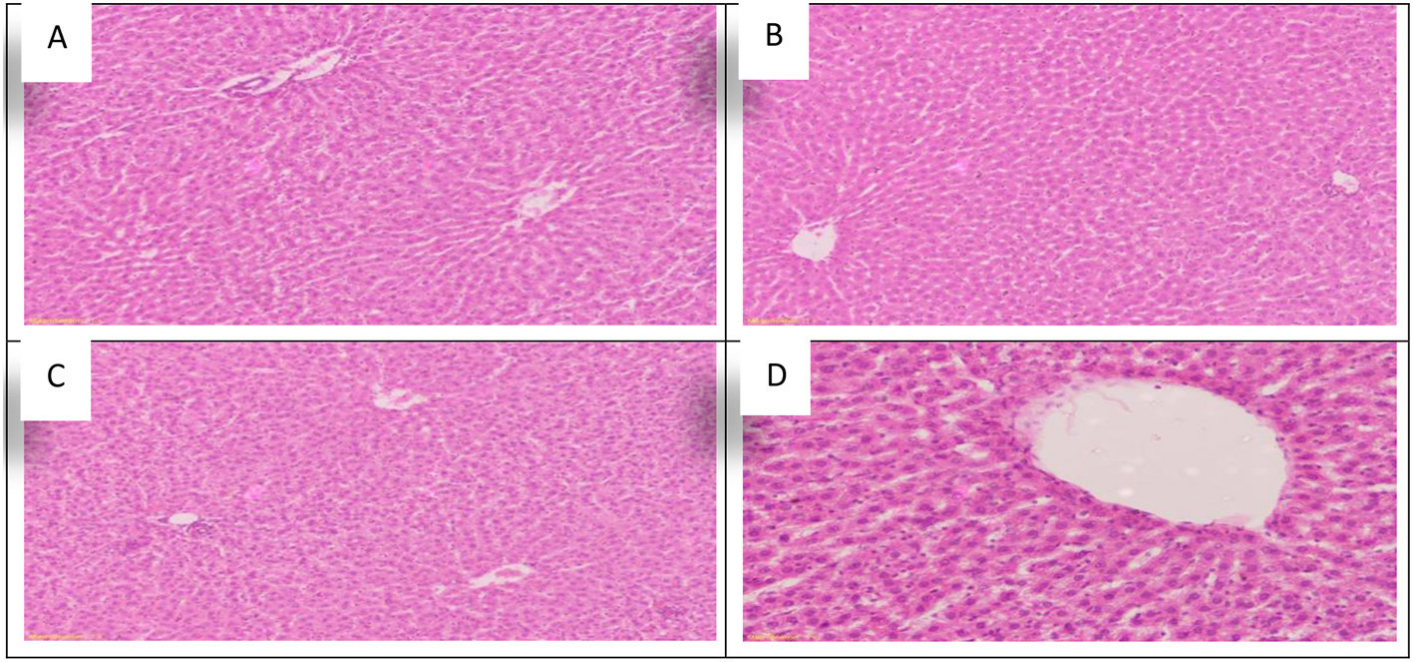
Schematic diagram showing the effect of each aluminum, medicinal plant, and mix with aluminum and medicinal plants on histopathology of rats' liver during return to normal after exposure to aluminum toxicity at 60 days after treatment. **(A)** Control group. **(B)** *Allium sativum* 5%. **(C)** *Nigella sativa* 5%, and **(D)** *Allium sativum* 2.5% + *Nigella sativa* 2.5%.

Using *Allium sativum* 5% and *Nigella sativa* 5%, and a mix of them (*Allium sativum* 2.5% + *Nigella sativa* 2.5%) to return the normal state after exposure to aluminum toxicity, as well as histopathology of rats' liver at the 16th week is shown in Figure 5.

The study obtained a positive effect due to *Allium sativum* and *Nigella sativa* in improving the harmful effects that the liver was exposed to during the treatment with aluminum. The optimum effect was the mixture between *Allium sativum* and *Nigella*, where liver tissue returned to its normal state (Figure 5D).

Sections in the liver tissue of all treated groups showed preserved lobular architecture. Also, the portal tracts consisted of the hepatic artery, portal vein, bile duct, and central veins; some were normal or congested. The hepatocytes were normal in arrangement, cytoplasm, and nuclei, and Congestion in the hepatic sinuses was not detected (Figures 5A–D).

#### Histopathological kidney

**Figure 6:**
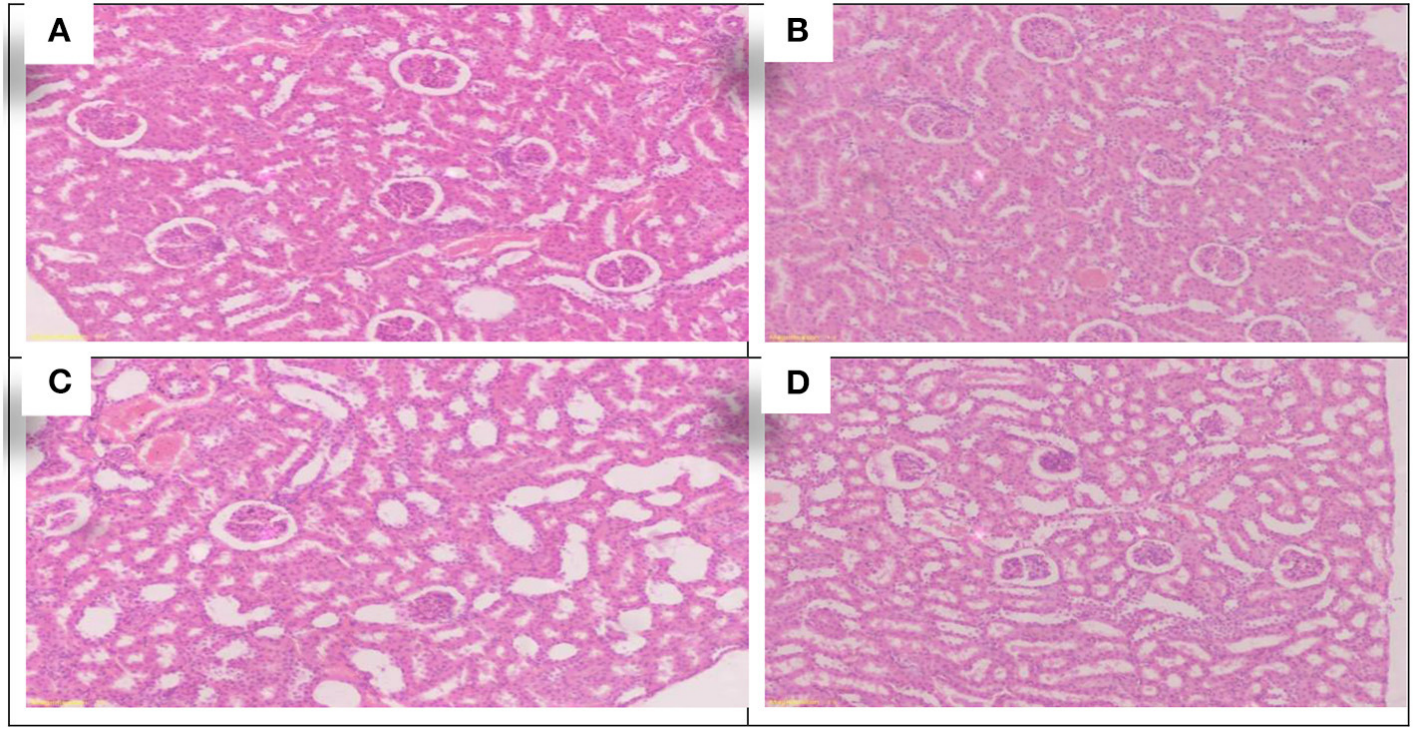
Schematic diagram showing the effect of each aluminum, medicinal plant and mix with aluminum and medicinal plants on histopathology of rats' Kidney during return to normal after exposure to aluminum toxicity at 60 day after treatment. **(A)** Control group. **(B)** *Allium sativum* 5%. **(C)** *Nigella sativa* 5%, and **(D)** *Allium sativum* 2.5% + *Nigella sativa* 2.5%.

Using *Allium sativum* 5% and *Nigella sativa* 5%, and a mix of them (*Allium sativum* 2.5% + *Nigella sativa* 2.5%) for return to normal state after exposure to aluminum toxicity, as well as histopathology of rats' kidneys at the 16th week, is shown in Figure 6.

The study found that the rats treated with *Allium sativum* 5% and *Nigella sativa* 5% had well-maintained kidney tissue structure, the cortex revealed normal glomeruli, tubules with minimal congestion in the *Allium sativum* 5% treated groups, and mild congestion in the *Nigella sativa* 5% treated groups of the cortical blood vessels. In addition, the medulla revealed normal tubules and minimal congested blood vessels of interstitial tissue in the *Allium sativum* 5% and average tubules and interstitial tissue in the *Nigella sativa* 5% treated groups (Figures 6B,C).

For the treatment with *Allium sativum* 2.5% plus *Nigella sativa* 2.5%, the results showed preserved architecture in kidney tissue; the cortex revealed normal glomeruli and tubules, while the medulla revealed normal tubules and minimal congestion of blood vessels of interstitial tissue (Figure 6D).

#### Histopathological testes

**Figure 7:**
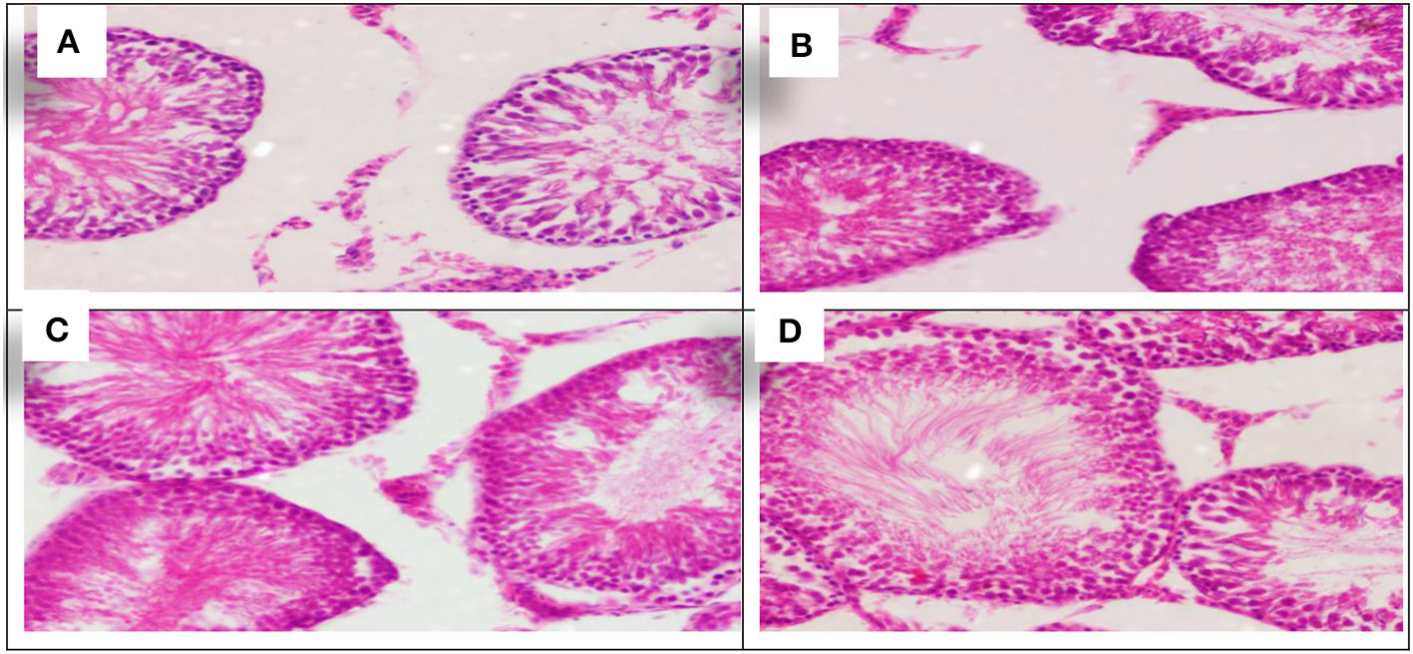
Schematic diagram showing the effect of each aluminum, medicinal plant, and mix with aluminum and medicinal plants on histopathology of rats' testicular tissues during return to normal after exposure to aluminum toxicity at 60 days after treatment. **(A)** Control group. **(B)** *Allium sativum* 5%. **(C)** *Nigella sativa* 5%, and **(D)** *Allium sativum* 2.5% + *Nigella sativa* 2.5%.

Using *Allium sativum* 5% and *Nigella sativa* 5%, and a mix of them (*Allium sativum* 2.5% + *Nigella sativa* 2.5%) for return to normal state after exposure to aluminum toxicity, as well as histopathology of rats' testes at the 16th week, are shown in Figure 7.

The sections showed similarity in all groups of testicular tissue, which showed preserved lobular architecture with normal seminiferous tubules and Sertoli cells. The testicular tissue showed preserved histological architecture, and the testicular seminiferous tubules consisted of normal basement membrane spermatogonia arranged in a stratified manner, spermatocytes and spermatozoa in the lumen. Sertoli cells were normal in number and arrangement (The sections showed similarity in all groups of testicular tissue, which showed preserved lobular architecture with normal seminiferous tubules and Sertoli cells. The testicular tissue showed preserved histological architecture, and the testicular seminiferous tubules consisted of normal basement membrane spermatogonia arranged in a stratified manner, spermatocytes and spermatozoa in the lumen. Sertoli cells were normal in number and arrangement (Figures 7A–D).

#### Serum blood biochemical parameters

The results of biochemical blood parameters in control, aluminum, *Allium sativum*, and *Nigella sativa* at the 16th week during return to normal after exposure to aluminum 1,600 ppm toxicity are presented in Table 2.

**Table 2:** Serum Biochemical Parameters (mg//dl) in control, aluminum, *Allium sativum*, and *Nigella sativa* at 16th week during exposure to aluminum 1,600 ppm toxicity (n = 5/group, mean ± SEM).

**Parameter**	**Time**	**Control**	***Alliumsativum* ^5%^**	**N. *Sativa*^5%^**	***Allium sativum* ^2.5%^ + N. *Sativa*^2.5%^**	**Sig**
Total	16th	8.80 ± 0.10^b^	8.14 ± 0.04^c^	9.14 ± 0.04^a^	8.14 ± 0.04^c^	***
Albumin	16th	5.77 ± 0.03^b^	5.27 ± 0.07^c^	5.87 ± 0.03^b^	6.17 ± 0.03^a^	***
Creatinine	16th	0.72 ± 0.01^a^	0.94 ± 0.02^a^	0.85 ± 0.03^b^	0.70 ± 0.01^a^	***
Urea	16th	34.33 ± 1.86^ab^	37.67 ± 1.33^a^	35.67 ± 1.20^ab^	31.33 ± 0.67^b^	*
ALT	16th	45.33 ± 2.03^b^	50.00 ± 0.58^ab^	51.33 ± 1.76^a^	45.67 ± 0.88^b^	**
AST	16th	30.46 ± 1.86^b^	31.76 ± 1.45^a^	31.36 ± 1.45^a^	30.23 ± 0.88^b^	***

a, b, c, d Means with different superscripts in the same row are *Means in the same row differ significantly (*p* ≤ 0.05); **Means in the same row differ significantly (*p* ≤ 0.01); ***Means in the same row differ significantly (*p* ≤ 0.001).

There was a significant increase in the total protein concentration (mg/dl; mean = SEM) of the rats treated with *Allium sativum* 5%, *Nigella Sativa* 5%, and a mix of the *Allium sativum* 2.5% plus *Nigella Sativa* 2.5% than the control group at 16th week. However, the Creatinine concentration (mg/dl; mean = SEM) in the 16th week had no significant changes among *Allium sativum* 5% and mix of the *Allium sativum* 2.5% plus *Nigella Sativa* 2.5% treated and control group, while the rats treated with *Nigella sativa*, 5% had a higher Creatinine concentration than other groups (Table 2).

For urea concentration (mg/dl; mean = SEM), no significant differences were found in the urea levels (mg/dl) among *Allium sativum* 5%, *Nigella sativa* 5%, and mix of the *Allium sativum* 2.5% plus *Nigella Sativa* 2.5% treated groups than Control group at 16th week. Also, the liver function enzymes showed no significant differences in the ALT and AST (mg/dl; mean = SEM) levels between the *Allium sativum* 2.5% mix plus *Nigella Sativa* 2.5% treatment and control group at the 16th week. While the *Nigella sativa* 5% treated group was significantly higher (*p* < 0.01) in the ALT and AST level (mg/dl; mean ± SEM) in comparison with the Control group (Table 2).

## Discussion

This experiment successfully evaluated the effect of *Nigella sativa* and *Allium sativum* supplementation on reducing the adverse effects of aluminum and returning the Albino Rats' liver, kidney, and testes to their natural state. Furthermore, in this study, *Allium sativum* and *Nigella sativa* were used during exposure to aluminum toxicity and after the aluminum treatment was discontinued.

The current study revealed adverse effects of exposure to aluminum where hepatic sinusoid congestion and the liver's central veins were normal in some places but congested in others. Following the treatments with *Nigella sativa* and *Allium sativum*, it found beneficial effects in avoiding the harmful effects of aluminum on liver tissues. Moreover, the rats treated with *Nigella sativa* showed normal liver tissue, the preserved lobular architecture; the portal tracts consisted of the normal hepatic artery, portal vein, and bile duct, and the hepatocytes were normal in an arrangement of cytoplasm and nuclei. *N. Sativa* administration protects hepatic tissue from the deleterious effects of toxic metals (27). In addition, *N.sativa* provided cytoprotectant by improving the histoarchitecture of the liver and decreasing the number of apoptotic cells (28).

In rats with hepatic ischemia, biochemical parameters such as total oxidative status and oxidative stress index were measured in hepatic tissue. The results indicated that *N. sativa* treatment protects the rat liver from hepatic ischemia-reperfusion injury by increasing antioxidant enzymatic activities significantly (29). Furthermore, Through its antioxidant and anti-apoptotic properties, *N. Sativa* was effective in protecting against hepatotoxicity (28).

Several studies showed that *Allium sativum* could protect the liver cells from some toxic agents. Dietary inclusion of *Allium sativum* powder protects against rat hepatotoxicity, improves antioxidant status, and modulates oxidative stress (30). In comparison, the co-administration of leaves' wild garlic has reduced histological injuries in the hepatic and renal rats (31). In addition, *Allium sativum* attenuated the hepatotoxicity effect of nitrate in rats. *Allium sativum* extract may reduce lipid peroxidation and enhance the antioxidant defense system (32). Another study suggested that *Allium sativum* with a high dose has the potential ability to induce liver damage (33).

The above results on the Kidney section revealed that rats treated with *Nigella sativa* produced normal Kidney samples, with sections of kidney tissue displaying preserved architecture. Normal glomeruli and tubules were found in the cortex, and normal tubules were found in the medulla. This indicates that *Nigella sativa* had positive effects on evading the harmful effects of aluminum in Kidney tissues. The protective effect of N. Sativa against tubular necrosis in rat kidneys was practical; it also found a significant reduction in total oxidative status and oxidative stress index levels in kidney tissue (34, 35). Furthermore, N. Sativa acts in the kidney as a powerful free radical scavenger, preventing the toxicon both biochemical and histopathological parameters (36).

Albrakati et al. (37) reported that *Allium sativum* extract supplementation significantly increased renal structure and function changes, indicating its renoprotective effect by protecting cellular membrane integrity. However, it did not show any change in the kidney histology compared to the control group in the rats treated with *Allium sativum* extract, according to Shiju et al. (38).

The testicular tissue showed preserved histological architecture, and the testicular seminiferous tubules consisted of normal basement membrane spermatogonia arranged in a stratified manner, spermatocytes and spermatozoa in the lumen. Sertoli cells were normal in number and arrangement. Most of the therapeutic properties of the *Nigella sativa* are due to the presence of thymoquinone (TQ) and its protective role on testicular toxicity in male rats; as they monitor decreased total antioxidant capacity and finally, administration of TQ may decrease the destructive effects of testicular tissue (39).

Testicular tissue is susceptible to free radicals and oxidative stress because the tissue has a high cell division rate. It was postulated that increases in oxidative stress could reduce testosterone secretion by testicular Leydig cells (40). Also, Lotfi et al. (41) concluded that the *Allium sativum* could help to reduce the severity of damage in the testicular tissues of diabetic rats through its hypoglycemic, antioxidant, and anti-inflammatory properties; *Allium sativum* extract was associated with reduced glucose levels, oxidative stress, interleukin-1 β levels, and gene expression of inducible nitric oxide synthase and increased testosterone levels and sperm quality.

In addition, the biochemical measurements ensure that the *Nigella sativa* and *Allium sativum* supplementation could reduce the adverse effects of aluminum and return the Albino Rats' liver, kidney, and testes to their natural state. There were no significant differences between *Allium sativum* 5% and *Nigella sativa* 5% in the total protein concentration. The animals treated with a mix of aluminum and medicinal plants showed that total protein concentration approached the control group. These results follow those of Al-Logmani and Zari (42). They reported that treatment of diabetic and non-diabetic rats with 5% *Nigella sativa* oil for seven weeks did not significantly affect the serum concentration of total protein as compared with untreated diabetic and control rats. Ghalehkandi et al. (43) showed that treatment of rats with *Allium sativum* aqueous extract had no significant changes in the serum value of total protein. Also, they found that combinative use of *Allium sativum* and CrCl3 does not affect the serum value of total protein.

In the present study, the serum creatinine and urea concentration were measured as indicators of nephrotoxicity for all the groups of rats. The serum creatinine and urea levels were very close between animals treated with *Nigella sativa* and the control group. Abul-Nasr et al. (44) revealed that *N. Sativa* had a nephroprotective effect as they lowered the values of serum creatinine and urea; also, N. *Sativa* had protective effects on albino rats. Estimating urea and creatinine reflects kidney function status, and their elevation is associated with renal impairment (45).

Treatment with *Allium sativum* and Nigella for liver enzyme function decreased ALT and AST percentage after the 8th week of aluminum exposure. No significant differences were reported in the AST and ALT concentrations among *Allium sativum, Nigella sativa*, and control groups. Hassanin and Hassan (46) found no significant effect on serum transaminases (AST and ALT) in male guinea pigs treated with *Nigella sativa*. At the same time, Al-Jishi and Haifa (47) showed that *Nigella sativa* seed powder (180 mg/kg rat/day) increased ALT activity, but no changes were noticed in AST activity in rats. In addition, Samson et al. (48) showed a significant decrease in serum ALT and AST compared with the control group in the rats treated with *Allium sativum* (200, 400, and 600 mg/kg/day).

It was noted that the feed intake in the group treated with *Allium sativum* was lower than the rest of the groups due to the lack of palatability for the rat to eat *Allium sativum*, and this made the mixture better in results, in addition to an improvement in the proportion of feed intake. This may be explained by garlic and Nigella's potential as a natural antioxidant, in addition to the ability of garlic to activate liver enzymes that help the body to get rid of toxins. Concerning kidney functions, as the percentage of fibrosis decreased and there was an improvement in the structures of the liver and kidneys, the black bean works to speed up the mechanism of protecting the liver and kidneys. The black seed also inhibits hepatic and renal toxicity.

## Conclusion

In conclusion, aluminum has adverse effects on animal and human health. Treatment with *Nigella sativa* and *Allium sativum* reduces aluminium's negative consequences and helps the Liver, Kidney, and Testes return to their natural state. Moreover, the blood biochemical findings in the present study ensured the efficacy of *Nigella sativa* and *Allium sativum* on aluminum toxicity. Furthermore, diets rich in medicinal plants (*Allium sativum* and Nigella) could be beneficial in alleviating aluminum toxicity.

## Data availability statement

The raw data supporting the conclusions of this article will be made available by the authors, without undue reservation.

## Ethics statement

This study was reviewed and approved by Animal Production Department, Faculty of Agriculture, Al-Azaher University, Cairo, Egypt (no: ASS; 2022/05/1161).

## Author contributions

Conceptualization, project administration, funding acquisition, and supervision: MEA, SA, and MAM. Methodology and writing—review and editing: MEA, MA, YSM, and AA-D. Formal analysis: FA-S, AA-D, MA, and AEA. Investigation: MEA, SA, MAM, and MA. Writing—original draft preparation: MEA, AA-D, and MA. All authors have read and agreed to the published version of the manuscript.

## Conflict of interest

The authors declare that the research was conducted in the absence of any commercial or financial relationships that could be construed as a potential conflict of interest.

## Publisher's note

All claims expressed in this article are solely those of the authors and do not necessarily represent those of their affiliated organizations, or those of the publisher, the editors and the reviewers. Any product that may be evaluated in this article, or claim that may be made by its manufacturer, is not guaranteed or endorsed by the publisher.
