# Corrigendum: Effect of *Allium sativum* and *Nigella sativa* on alleviating aluminum toxicity state in the albino rats

**DOI:** 10.3389/fvets.2023.1160163

**Published:** 2023-03-09

**Authors:** Sayed Soliman Abdel Ghfar, Montaser Elsayed Ali, Maha Abdullah Momenah, Fatimah A. Al-Saeed, Amin A. Al-Doaiss, Yasser Sabry Mostafa, Ahmed Ezzat Ahmed, Mohamed Abdelrahman

**Affiliations:** ^1^Department of Animal Productions, Faculty of Agriculture, Al-Azhar University, Cairo, Egypt; ^2^Department of Animal Productions, Faculty of Agriculture, Al-Azhar University, Assiut, Egypt; ^3^Department of Biology, College of Science, Princess Nourah bint Abdulrahman University, Riyadh, Saudi Arabia; ^4^Biology Department, College of Science, King Khalid University, Abha, Saudi Arabia; ^5^Department of Theriogenology, Obstetrics, and Artificial Insemination, Faculty of Veterinary Medicine, South Valley University, Qena, Egypt; ^6^Key Lab of Agricultural Animal Genetics, Breeding and Reproduction of Ministry of Education, Huazhong Agricultural University, Wuhan, China; ^7^Animal Production Department, Faculty of Agriculture, Assiut University, Asyut, Egypt

**Keywords:** aluminum toxicity, *Allium sativum*, *Nigella sativa*, histopathological abnormalities, liver, kidney, testes

In the published article, there was an error in affiliations 4, 5, 6, 7. Instead of:

“^4^Department of Biology, College of Science, King Khalid University, Abha, Saudi Arabia, ^5^Department of Theriogenology, Faculty of Veterinary Medicine, South Valley University, Qena, Egypt, ^6^Key Lab of Agricultural Animal Genetics, Breeding and Reproduction of Ministry of Education, Huazhong Agricultural University, Wuhan, China, ^7^Animal Production Department, Faculty of Agriculture, Assuit University, Asyut, Egypt”

The affiliation numbers of these authors should be:

“Fatimah A. Al-Saeed^4^, Amin A. Al-Doaiss^4^, Yasser Sabry Mostafa^4^, Ahmed Ezzat Ahmed^4,5^ and Mohamed Abdelrahman^6,7^”

And affiliations 4, 5, 6 and 7 should be:

“^4^Biology Department, College of Science, King Khalid University, Saudi Arabia, ^5^Department of Theriogenology, Obstetrics, and Artificial Insemination, Faculty of Veterinary Medicine, South Valley University, Qena, Egypt, ^6^Key Lab of Agricultural Animal Genetics, Breeding and Reproduction of Ministry of Education, Huazhong Agricultural University, Wuhan, China, ^7^Animal Production Department, Faculty of Agriculture, Assiut University, Asyut, Egypt”

The authors apologize for this error and state that this does not change the scientific conclusions of the article in any way. The original article has been updated.

